# A Methodology for Detecting Field Potentials from the External Ear Canal: NEER and EVestG

**DOI:** 10.1007/s10439-012-0526-3

**Published:** 2012-02-09

**Authors:** Brian Lithgow

**Affiliations:** 1 Monash Alfred Psychiatry Research Centre, The Alfred Hospital, Commercial Rd., Room I43 Old Baker Building, Prahran, Australia; 2 Electrical & Computer Engineering Department, University of Manitoba, Winnipeg, MB R3T5V6 Canada; 3 Diagnostic and Neurosignal Processing Research Laboratory, Riverview Health Center, Room PE456, Admin Bldg., 1 Morley Ave., Winnipeg, MB R3L2P4 Canada; 4 Diagnostic and Neurosignal Processing Research Laboratory, Electrical and Computer Systems Engineering, Monash University, Wellington Rd., Clayton, VIC Australia

**Keywords:** Vestibular, Diagnostic, ECOG, Balance

## Abstract

An algorithm called the neural event extraction routine (NEER) and a method called Electrovestibulography (EVestG) for extracting field potentials (FPs) from artefact rich and noisy ear canal recordings is presented. Averaged FP waveforms can be used to aid detection of acoustic and or vestibular pathologies. FPs were recorded in the external ear canal proximal to the ear drum. These FPs were extracted using an algorithm called NEER. NEER utilises a modified complex Morlet wavelet analysis of phase change across multiple scales and a template matching (matched filter) methodology to detect FPs buried in noise and biological and environmental artefacts. Initial simulation with simulated FPs shows NEER detects FPs down to −30 dB SNR (power) but only 13–23% of those at SNR’s <−6 dB. This was deemed applicable to longer duration recordings wherein averaging could be applied as many FPs are present. NEER was applied to detect both spontaneous and whole body tilt evoked FPs. By subtracting the averaged tilt FP response from the averaged spontaneous FP response it is believed this difference is more representative of the vestibular response. Significant difference (*p* < 0.05) between up and down whole body (supine and sitting) movements was achieved. Pathologic and physiologic evidence in support of a vestibular and acoustic origin is also presented.

## Introduction

**Figure 1:**
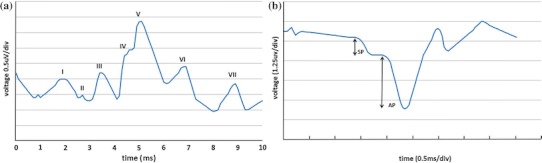
(a) Typical ABR response (note: ABR waveform is by convention inverted relative to the ECOG waveform); (b) typical ECOG (SP/AP) response shape

When hair cells in the inner ear are bent (e.g., through sound vibration for hearing or body movement for maintaining balance), through a process of mechanical to electrical transduction, this can lead to individual nerve spikes (electrical activity). When a group of hair cells are bent, contributions from the group of neurons may overlap, producing extracellular potentials. If they are large enough, these signals are commonly called field potentials (FPs). Normally, the recorded signals immediately after the driving stimulus are averaged to produce a waveform used to assist in the diagnosis of certain pathologies. Averaging is necessary to generate a diagnostically useful output waveform as the FPs are commonly buried in a sea of noise (EEG-brain, EOG-eye, power line, EMG-muscle, etc.). Examples of averaging evoked FPs to produce a diagnostic waveform include the Electrocochleography (ECOG)^9^,^10^ and the auditory brainstem response (ABR).^5^ The ABR (Fig. 1a) is an auditory evoked potential recorded with scalp electrodes; it consists of seven main peaks, each of which represents nuclei along the auditory pathway and whose presence and relative position provide measures of the intactness and functionality of the auditory pathway.^5^ The ECOG (Fig. 1b) is a variant of the ABR focusing on the first peak of the ABR, wherein the active electrode is placed very close to the tympanic membrane^9^,^10^; It is commonly used to diagnose vestibular problems especially Meniere’s Disease.^9^,^10^ The ECOG is interpreted by comparing the height of the summing potential (SP) to the action potential (AP) (Fig. 1b).^9^,^10^ Both the ECOG and ABR methodologies typically require more than 300 acoustic driving stimuli. While the ABR uses an acoustic input to measure the acoustic systems’ functionality, the ECOG, uses the acoustic stimuli to generate an indirect measure of the peripheral vestibular system. On each side of the skull, the peripheral vestibular system is made up of the utricle and saccule, which measure linear and vertical acceleration, plus the three orthogonally oriented semi circular canals that sense rotation. A more direct ECOG-like measure of vestibular FPs may be clinically more useful given that the ECOG has for diagnosing Meniere’s Disease an accuracy of 85% for the invasive trans-tympanic recording,^15^ and correct classification rate of 64–74% for the non-invasive extra-tympanic recording.^21^

An alternative method would be to use a direct vestibular stimulus such as a whole body tilt. However, a major limitation to multiple stimulation and subsequent averaging of a direct vestibular stimuli is the long time before the vestibular system returns to equilibrium. The times often cited are of the order 20–30 s.^7^ This implies the required time to apply the minimum 300 stimuli in order to have a representative averaged response, is more than 2.5 h for each component of the vestibular periphery; therefore, the total required time to test the three SCCs, utricle and saccule would be more than 12.5 h which is unrealistic. Faster invasive methodologies exists and have been evaluated in animals. For example, in guinea pigs, vestibular evoked potentials (VsEPs) have been recorded in response to 400 alternating vertical acceleration pulses (4 g peak) delivered at a rate of 51/s.^20^ A separable vestibular component was produced. However, this method cannot be applied to humans as the head shaker was bolted to the skull and invasive electrodes used. The most common vestibular testing protocol for humans incorporates the Electronystagmography (ENG) and a rotational chair test, both of which only test the horizontal semi circular canal (SCC).^25^ ENG or caloric testing is the response to hot and cold water (or air) in the ear canal, whilst the rotational chair test measures the eyes’ response as the chair is slowly rotated. The accuracy of ENG to detecting nerve damage as a cause of vertigo is suggested to be about 80%,^1^ whilst the accuracy of rotational chair testing is disputed based on the many protocols applied. Newer methods such as sound evoked myogenic potentials (VMEP) and 3D video-oculography (with unilateral centrifugation) are being used to assess predominantly saccule and utricle function, respectively.^4^,^11^ Video-oculography is akin to ENG using video rather than electrodes to record the response.^11^ Using only one methodology, such as Electrovestibulography (EVestG),^19^ to non-invasively, unilaterally and directly test each component of the vestibular system by detecting a train of FPs in response to a single stimulus in each plane would be advantageous. It is known that there is spontaneous activity in hair cells of the auditory and vestibular systems and that spontaneous activity can lead to the generation of FPs.^3^,^12^,^23^ This paper presents an algorithm, the neural event extraction routine (NEER), for detecting spontaneous and driven FPs recorded in the external ear canal by EVestG.^19^ No algorithm could be found in the literature that successfully detects individual spontaneously evoked vestibular FPs using an ear canal electrode. NEER utilises a modified complex Morlet wavelet analysis of phase change across multiple scales and a template matching methodology (matched filter) to detect FPs buried in noise and biological and environmental artefacts. The signals recorded by EVestG are similar to those of ECOG. Jones *et* *al*.^16^ has shown that the FP shape of the recorded signal from mice, proximal to elements of the vestibular periphery, is very similar to that generated by the ECOG technique. Therefore, the NEER algorithm is also using a similar FP matching template technique to extract FPs buried in noise from the recorded signals.

The first hypothesis is that the NEER algorithm can detect FPs at negative SNRs with a high enough detection rate to enable generation of SP/AP like plots (Fig. 1b) for vestibular signals. In this study, the signals incorporating the FPs were recorded in both background and in response to a passive whole body tilt. The FPs were then averaged to produce a characteristic waveform for resting and tilted scenarios. During a whole body title, both vestibular and acoustic hair cells are spontaneously active. The active ear canal recording electrode is proximal to both acoustic and vestibular sources, and likely to contain both acoustic and vestibular input. Given the acoustic input is minimally affected by “quiet” whole body tilts the second hypothesis is that by subtracting the resting response from the tilt response, the acoustic response will be further reduced and the resultant signal will be more representative of the vestibular response. These hypotheses are tested by comparing the output waveform for a group of orthogonal movement stimuli in a population 30 healthy controls. Furthermore, as another means to validate the hypotheses, the effect of nerve deafness and Labyrinthectomy on the EVestG output signals are examined.

## Methodology

In this section the following are sequentially described: (1) the EVestG recording methodology; (2) the NEER algorithm; (3) the method for testing the NEER algorithm with simulated FPs buried in noise with different SNRs, and (4) the testing of unilateral nerve deaf and Labyrinthectomy subjects.

### EVestG Recording

Data were collected from 30 healthy control subjects (ages 20–67, avg. = 39, SD = 15.8) with normal balance and hearing and not on any medication; data were recorded using EVestG methodology^19^ during three different movement stimuli. Ethics approval (95/06) was obtained from the Alfred Hospital, and all participants gave written consent prior to the experiments. The EVestG methodology is identical to ECOG^9^,^10^ with the exceptions that (1) the acoustic stimuli (multiple clicks or tones) are replaced with a whole body tilt and (2) the reference lead which is normally on the contralateral earlobe is applied to the ipsilateral earlobe to reduce interference from other proximal neurological sources.

**Figure 2:**
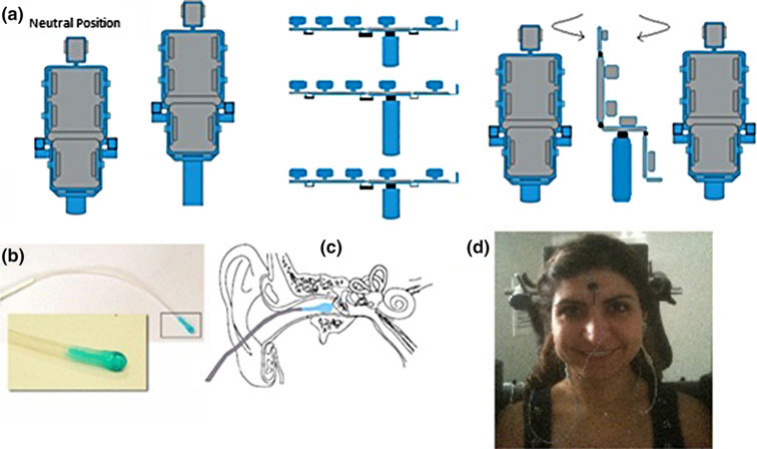
(a) Sitting up/down movement, supine up/down movement, sitting horizontal rotation. (b) Ear electrode; (c) electrode placement; (d) subject connections

**Figure 3:**
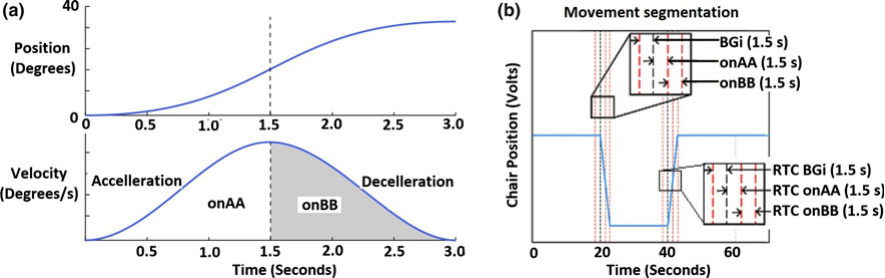
(a) Chair velocity profile during motion. The same profile was used for return to centre for each movement. (b) Movement segmentation definitions

EVestG recording: The subjects sat on a hydraulic chair with their eyes closed and head rested on the chair headrest; they received three passive whole body movement stimuli: moving up/down in both sitting upright and supine positions, and for a horizontal rotation to the right and return to center (RTC) whilst sitting upright. Figure 2 shows the recording apparatus. The recordings were made in an acoustically attenuated (>30 dB) and electromagnetically shielded chamber. The chair movement velocity profile is shown in Fig. 3a. The active gelled recording electrodes (Fig. 2b) were TM-EcochGtrode (Bio-logic, France) and placed in both ears proximal to the ear drum (Fig. 2c). Reference electrodes (Biopac EL254S for earlobe and EL258S for forehead) were placed on the ipsilateral earlobes and a common ground electrode was placed on the forehead (Fig. 2d).

The left and right ears’ signals were recorded using Spike2 software *via* a CED-1902 amplifier (50 Hz notch filter, 10 k gain, 1 Hz high pass filter) and digitized using CED1401 ADC board at sampling rate of 41,666 Hz. Each recording’s duration was 60 s for each stimulus: 20 s stationary at the centre position, 3 s motion to a position (up or rotated), 17 s stationary in that position, 3 s return to the centre, and another 17 s of stationary recording in the centre position.

The chair’s position was also recorded simultaneously with the ear signals; it was used to extract the segments of interest for analysis. The particular segments of interest are: 1.5 s immediately prior to the movement (BGi), 1.5 s acceleration (onAA) and 1.5 s deceleration (onBB) as well as the 1.5 s segments from the return to the centre: RTC BGi, RTC onAA and RTC onBB. These segments are shown on the chair’s movement signal in Fig. 3b. The acceleration/deceleration segments are selected as they give the largest differences compared to background.

Contamination with EEG remains possible but should be limited as the reference (earlobe) and active electrodes (tympanic membrane) are located close together. Signals contaminating the recording should be common to both static (BGi, RTC BGi) and movement phases (onAA, onBB, RTC onAA, RTC onBB); therefore, if poorly correlated with the vestibular response, when averaged over the typical 100–300 FPs they are expected to be removed or minimized. The three whole body movements were selected to emphasise, predominantly, the responses of the saccule (sitting up/down movement), utricle (supine up/down movement) and horizontal SCCs (horizontal rotation while sitting upright). The saccule predominantly detects vertical accelerations, while the utricle detects predominantly horizontal accelerations and the horizontal SCCs rotations in the horizontal plane. These movements also have the advantage of limiting hemodynamic effects by keeping the body in one orientation; thus, not requiring the subject to overly utilize new muscles to maintain a new whole body position.

After the recordings, the active ear electrodes are removed from the ears, the background noise is recorded and saved. Then, each of the left and right ear recorded signals along with the background noise are fed into Matlab using the SON library^18^ to the NEER algorithm to then extract the FPs; this is explained in the following section.

### Neural Event Extraction Routine Algorithm

**Figure 4:**
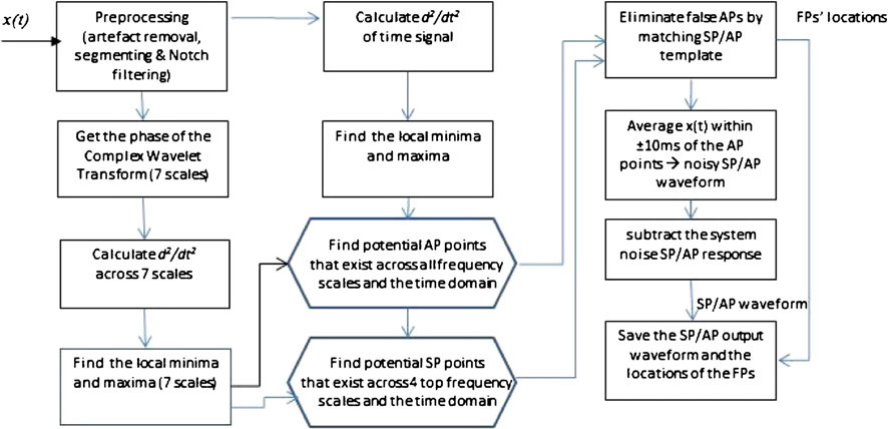
NEER algorithm flow chart

The NEER algorithm utilises a modified complex Morlet Wavelet Transform^13^ analysis of phase change across multiple scales and a template matching methodology (matched filter^24^) to detect FPs buried in noise and biological and environmental artefacts. Both Wavelet analysis and matched filtering are required as: (1) wavelet analysis of phase change across multiple scales finds the potential FPs but also a wide range of other neural signals, i.e., EEG, EMG, EOG, etc. due to the negative SNR, and (2) at negative SNRs the matched filter technique—if applied alone—will find the required pattern simply by chance but such a filter can provide some selectivity to the wide range of the detected neural signals by the wavelet analysis; therefore the combination of wavelet analysis and matched filter will improve the FPs detection. The NEER algorithm’s main FP detection module is similar to matched filtering, in which it uses the SP/AP template (Fig. 1b) to find the locations of the FPs. The matched filter is the optimum linear filter for extraction the signal of interest in the presence of additive stochastic noise.^23^ The NEER algorithm is shown schematically in Fig. 4, and is explained as the following steps; *x*(*t*) represents the recorded raw signal.

#### Preprocessing

- Large artefacts (if any) are removed using simple thresholding of mean plus or minus five standard deviations (*μ* ± *5*σ) of the recorded raw voltage signal. These artefacts are usually due to body movement and muscle contraction.
- Artefact free signals are passed through 120 and 25 Hz highpass zero phase filters^17^; hereafter, they are called *x*_120_(*t*) and *x*_25_(*t*).
- Using the chair’s position signal, extract the six segments (BGi, onAA, onBB, RTC BGi, RTC onAA and RTC onBB) from each of *x*_120_(*t*) and *x*_25_(*t*); they are referred to as seg_25_^*i*^ and seg_120_^*i*^, where *i* is one of the six segments.
- Pass each segment’s signal to the zero phase notch filters^17^ to remove power line harmonics and hydraulic chair artefacts. To do that the power spectral density (psd) of each segment is calculated and searched for peaks related to hydraulic artefacts. The threshold for notch filtering is set to approximately 6 dB above the average background. If these detected peaks corresponded to a hydraulic artefact frequency (typically 927 Hz—due to the hydraulic dither signal) the magnitude and width of the peak above the background level is calculated. Using this magnitude and the minimum possible filter width, a zero phase notch filter is applied. The width of the notch filter could be adjusted if the peak is wide.

#### Time–Frequency Analysis

- Each segment’s cleaned signal is then input to a Modified Complex Morlet Wavelet analysis module. The complex Morlet wavelet is defined by $\psi (x) = \frac{1}{{\sqrt {\pi f_{\text{b}} } }}e^{{2i\pi f_{\text{c}} x}} e^{{ - \frac{{x^{2} }}{{f_{\text{b}} }}}},$ where, *f*_b_ is the bandwidth factor, and *f*_c_ is a wavelet center frequency.^13^ The modification applied was to reduce the bandwidth factor from 1 to 0.1 and 0.4 for low and high frequency ranges, respectively; this was done to improve temporal resolution particularly for the low frequency scales. Seven scales set at approximately mid band frequencies *f*_c_ of {600, 900, 1200, 1500, 3000, 6000, 9000} are analysed. The phase is unwrapped and the mean value removed for each scale. Then, the 2nd derivative of the phase of the ψ(*x*) of each scale is calculated and a search made for sharp changes as these are potential FPs. Maxima of the 2nd derivative of the phase are located for each scale.
- After 120 Hz zero phase filtering^17^ the 2nd derivative of each segment’s time-domain signal is also calculated and searched for FPs.

#### FP Detection Module

- For the 25 Hz high pass recordings search for AP points in the 2nd derivative phase signals across all seven frequency scales and also in the 2nd derivative of the time-domain signal by identifying all the sharp changes (large local maxima) in the signals. If a sharp change exists across all the scales within a predetermined number of samples and also in the time-domain signal within a predetermined number of samples, then that sharp change is considered as a potential AP point.
- Search for SP points in the 2nd derivative phase signals across the four top frequency scales by identifying all the sharp changes (large local maxima) proximal to the potential AP loci.
- Match the potential AP and SP points with the SP/AP template (see Fig. 1b; for example, the SP points should be around 15–30 samples prior to AP and with lower magnitude in time-domain signal). This procedure eliminates many false SP and AP detected points and marks the loci of the AP points of the matched ones.
- Search for baseline points in the 2nd derivative phase signals across the four top frequency scales. This is done by identifying all the sharp changes (large local maxima) within a predetermined range based on the SP/AP template prior to the SP point and after the potential AP point and with both baseline points having lower magnitude in time-domain signal. This procedure also eliminates many false AP detected points and marks the loci of the AP points of the matched ones.
- Save the locations of APs as the FP loci. Loci, wherein individual FP waveforms overlap, are excluded.

#### Averaging, Denoising and Forming SP/AP Output Waveform

**Figure 5:**
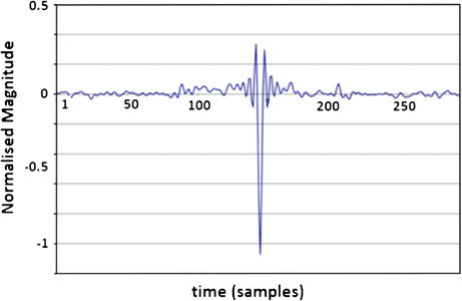
System noise response
- Save the cleaned SP/AP waveform and the locations of the AP points as the FPs loci as the system’s outputs.

- Average every 20 ms of the signal (seg_25_^*i*^) that is around ±10 ms of the detected AP points. This results in the SP/AP waveform that still includes the (system) noise response.
- Using the recorded background (system) noise (typically pink noise), derive the SP/AP response from the background (system) noise signal (Fig. 5) and subtract a scaled version of that from the SP/AP waveform of previous step.

From the FPs’ loci, the time intervals between two FPs are then calculated for (seg_25_^*i*^) and (seg_120_^*i*^), and can be used for deriving the histogram of the FPs.

To produce a population average curve the SP/AP plots are normalised based on the largest of the response phases (typically the individuals onAA or onBB responses) before summing.

### Simulated FPs: Detection in Noise

**Figure 6:**
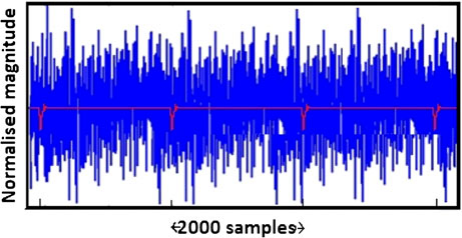
FP (red) plus noise (blue) segment of example test signal: synthetic FPs repeated each 2000 samples. Fs = 41,666

A simulated waveform based on Fig. 1b akin to an FP was generated in Matlab and embedded in a pre-recorded noise signal (Fig. 6). The pre-recorded noise signal was recorded while a subject was fully connected for an EVestG recording but with the active electrodes hanging out of the ear with all other conditions as for a recording; an artefact free segment was manually selected as the system noise, and was added to the simulated waveform. A total of 93 FP’s were spaced 2000 samples (~50 ms) apart. FP plus noise signals with SNR values from 0 dB in 3 or 6 dB steps down to −36 dB were generated, and were fed to the NEER algorithm to determine detection rate as a function of SNR. Only negative SNRs were evaluated as these are indicative of the SNR found in ear canal recordings.

### Tests on Nerve Deafness and Labyrinthectomy

As mentioned earlier, the recordings made in the ear canal are a mix of vestibular, acoustic and other artefact signals. In order to clarify the origin and nature of signals and relative acoustic and vestibular inputs, two particular pathologies were examined to separate vestibular and acoustic components. The EVestG signals were recorded in response to the same movement stimuli in two patients: one with nerve deafness in one side and another with a chemical Labyrinthectomy in one side.

- Patient 1 was nerve deaf in the right ear and normal hearing in the left; he did not have any vestibular problems. For this patient, the right side is considered as “pure” vestibular because the subject was nerve deaf on the right side. The left side’s response is expected to be a mix of vestibular and acoustic response.
- Patient 2 had a chemical Labyrinthectomy on his right side, and his left side vestibular was normal. Both ears of this patient had a similar high frequency hearing loss. The right side response is expected to be “pure” acoustic and the left side a mix of vestibular and acoustic response.

For these two patients, the static RTC BGi recordings were examined to simplify analysis and dynamic factors.

## Results

### Testing the NEER Algorithm with Simulated FP

**Figure 7:**
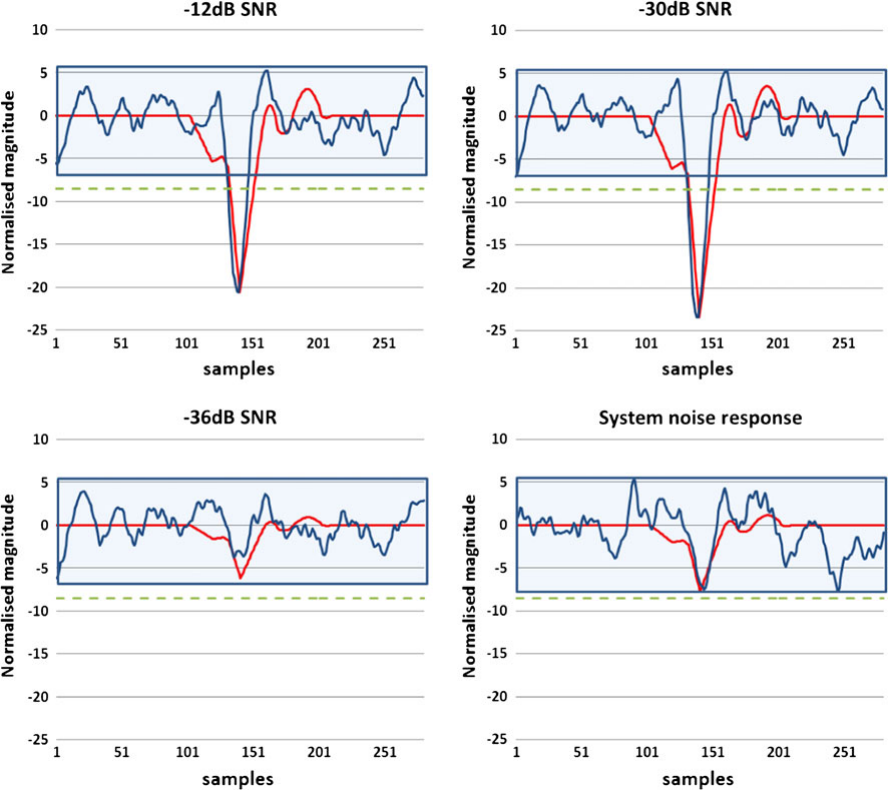
NEER output when detecting FPs hidden in various levels of noise. These figures clearly demonstrate the algorithms ability to find FPs down to −30 dB. Red = simulated FP. Blue = NEER output. Green dotted = detection threshold. Blue shade is the noise bed

**Figure 8:**
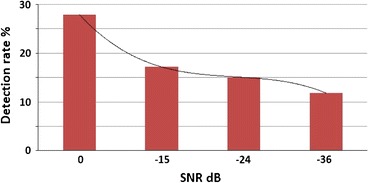
The NEER detection rate

Figures 7a–7d show the results of NEER algorithm on the simulated signal. It is apparent that FPs are detected down to −30 dB SNR. For worse SNR ratios the response approximates the system’s response to a noise only input, which is encouraging. Figure 8 shows the rate of detected FPs for the simulated signal.

As can be seen in Fig. 8, the major limitation of the NEER algorithm is its low detection rate. However the ability to find FPs with power levels down to −30 dB is encouraging enough to explore the algorithm’s ability to find real FPs in a difficult application, namely, vestibular FP detection using an ear canal electrode.

### Testing the NEER Algorithm on Vestibular Response Data (Control Population, *n* = 30)

**Figure 9:**
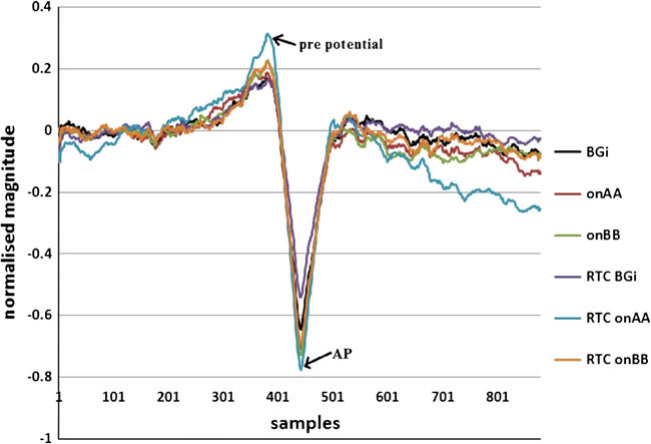
Normalised SP/AP plots for the supine up movement. Note: growth in AP and PPPs in moving from resting (BGi and RTC BGi) to motion (onAA, onBB, RTC onAA, RTC onBB)

Figure 9 shows the averaged SP/AP waveform extracted from the supine up/down data of the 30 control subjects. This movement has the least muscle movement, minimal hemodynamic response, and is considered the least likely to evoke an anxious response in the subjects. As can be seen in Fig. 9 one of the largest differences is between the RTC BGi and RTC onAA phases.

**Figure 10:**
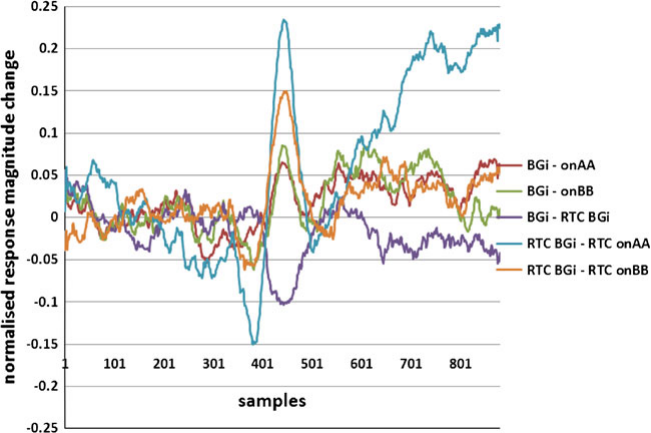
Normalised response change plots for moving from the static to moving frame for the supine up movement. Note: growth in AP and PPPs in moving from resting (BGi and RTC BGi) to motion (onAA, onBB, RTC onAA, RTC onBB). The transition from RTC BGi to RTC onAA gives the largest change in response

For the supine up/down motion, the downward movement is known to elicit the largest response.^6^,^20^ This is clearly observed in Fig. 9 and more clearly in Fig. 10, where the responses of motions are subtracted from the background. Again, the upward motion response change is smaller than the downward motion as expected.^8^ The acceleration phase (the RTC onAA segment) for a downward movement is the larger of the two large changes during downward motion (Fig. 10); this was expected because this response is predominantly utricular.

**Figure 11:**
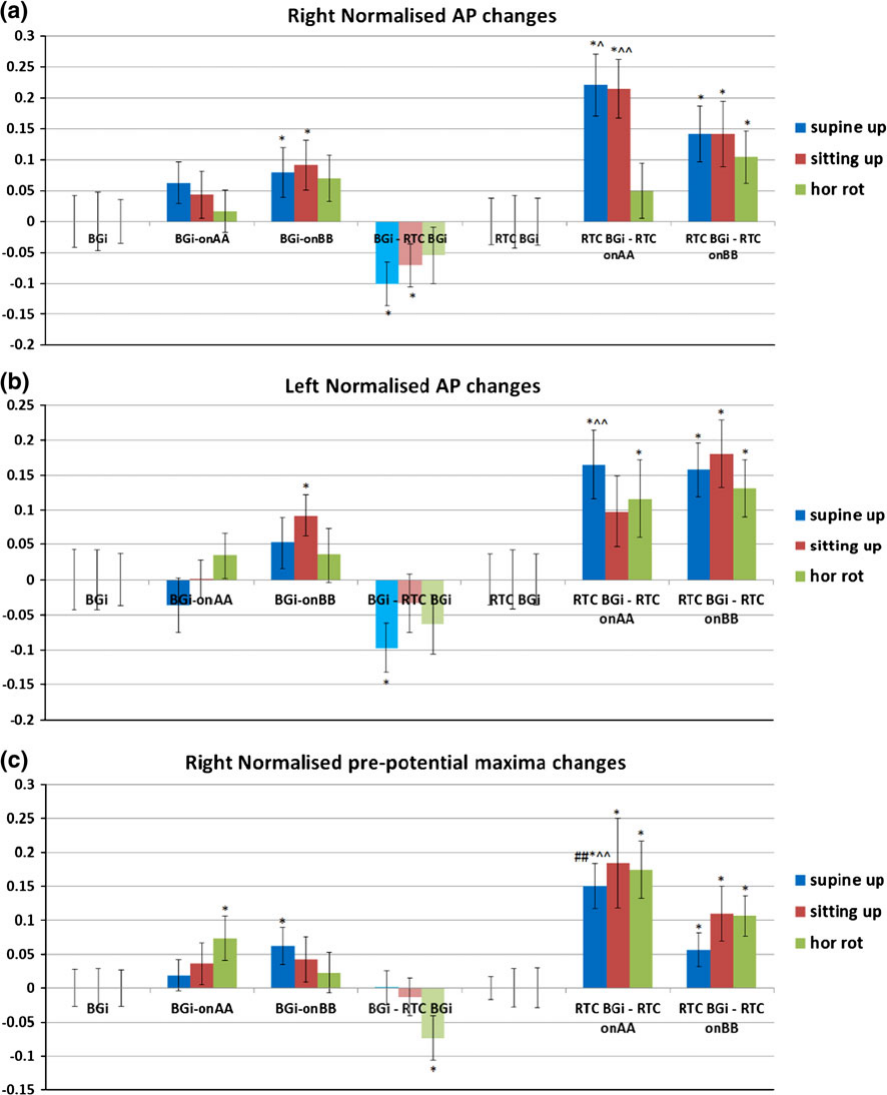

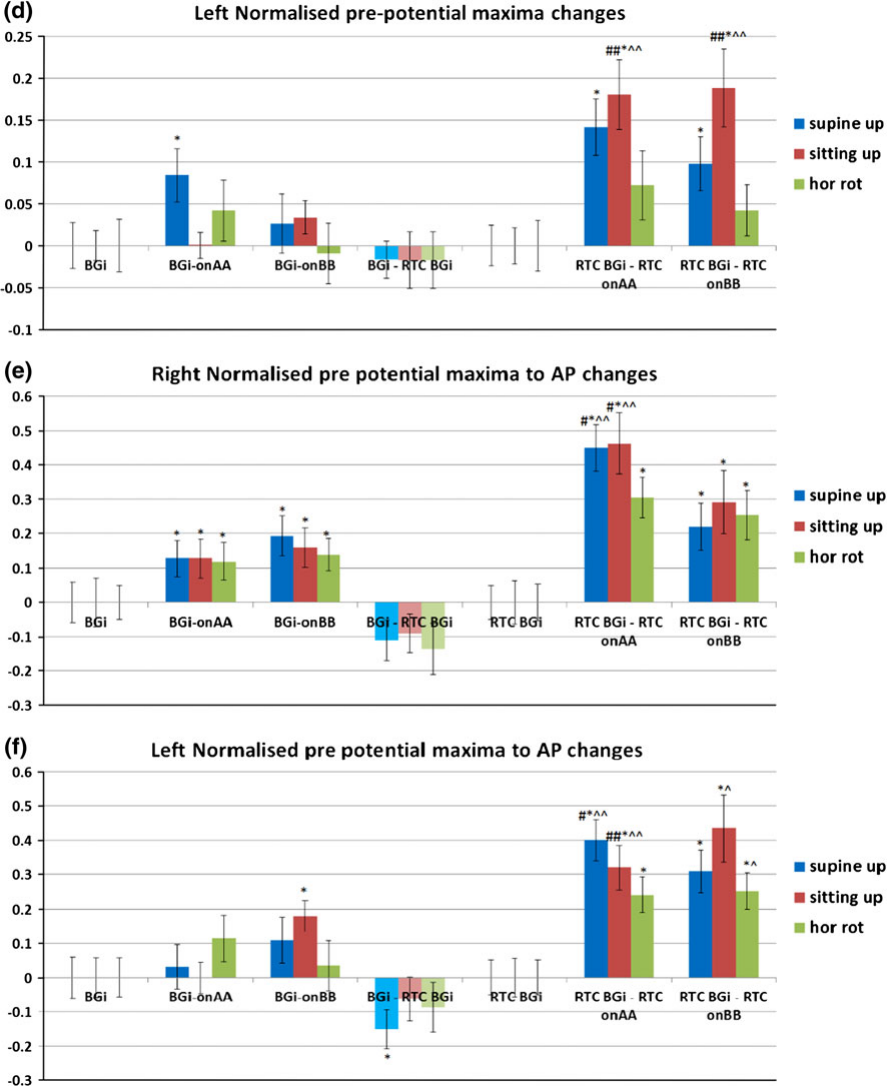
Right and left side changes in normalised AP magnitude (a, b), pre-potential magnitude changes (c, d) and pre-potential to AP magnitude changes (e, f) for a control population. *n* = 30. Standard error bars, *95% confidence difference (CD), ^90% CD between tilt and RTC segment (^^95% CD). ^#^90% CD between tilt and RTC segment after account for BGi–RTC BGi shift (^##^95% CD). The BGi response is chosen as the origin. RTC is the return to center segment response

**Figure 12:**
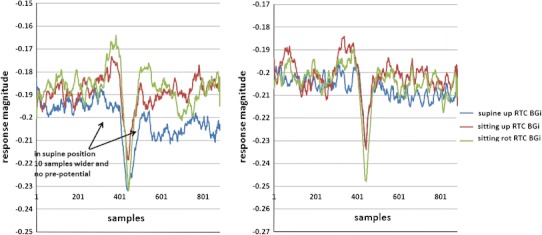
The SP/AP RTC BGi recording plots of the left side (left plot) (normal hearing) and the right side (right plot) (nerve deaf) of patient 1 who had no vestibular problems

Responses for other tilts/movements are considered in a similar manner. The normalized AP changes extracted from supine up/down (utricular), up (saccular) and horizontal rotation (horizontal SCC) movements for the right and left ears are shown in Figs. 11 and 12.

### Comparison of Up/Down, Supine Up/Down and Rotation Right/Left Performance

In the upright sitting position, the up/down movement evokes a predominantly saccular response with the downward movement being the strongest. A significantly larger AP change is observed for the downward relative to upward movement [e.g., when comparing the (RTC BGi–RTC onAA)–(BGi-onAA) changes] for right (onAA) and left (onAA and onBB) sides. For the right onAA movement the *p*-values of the difference was <0.05. For the right onAA response these differences remained significant even after accounting for any (BGi–RTC BGi) shift in AP size.

With the exception of the left BGi-onAA response, all left and right AP sizes were significantly different from that of the background (measured immediately prior to the movement). More specifically, with the exceptions of the left and right (BGi-onAA) and left (RTC BGi–RTC onAA) responses, all *p*-values were <0.05. Given that hemodynamic and muscle effects are limited in the sitting position, this is a good evidence for a vestibular (otolithic) response.

The supine up/down movement evokes a predominantly utricular response with the downward movement being the strongest. Therefore, mirror-reflected results as the above were expected to be seen. As above, a significantly larger AP change was observed for the downward relative to upward movement for right (onAA) and left (onAA and onBB) sides. For the right and left onAA movement *p*-values were less than 0.1 and 0.05, respectively. Also similar to that described above, with the exception of the left BGi-onAA response all left and right AP sizes were significantly different from that of the background (measured immediately prior to movement). More specifically, with the exceptions of the left and right (BGi-onAA) and left (BGi-onBB) responses, all *p*-values were <0.05. Given hemodynamic effects and muscle effects are minimal in the supine position, again this is a further evidence for a vestibular (otolithic) response.

Horizontal rotation evokes a predominantly horizontal SCC response with a toward ear rotation normally evoking the strongest response.^12^ Rotation is to the right first then return to left. A significantly larger AP value is found on the left side for the return to center (RTC onAA and RTC onBB phases) rotation i.e., towards the ear. This observation was seen on the right ear signal but only during the return deceleration (RTC onBB) phase i.e., not during the right rotation. This could be due to: (1) The head was not tilted forward at 30 degrees so the horizontal SCC was not perfectly in the plane of rotation meaning other parts of the vestibular system may have been stimulated, (2) Horizontal rotation is known to evoke a small utricular response,^14^ and if utricular these authors predict otolithic responses to be more than 10 times those of the SCC thus confounding simple interpretation. It may also be argued that the baseline shift (BGi–RTC BGi) is representative of a change in initial condition that can affect response sensitivity. Given haemodynamic effects and muscle effects are minimized in the sitting position this is evidence of a smaller vestibular response.

Comparatively with the AP point the pre-potential point (PPP) for the sitting up/down movement should evoke the strongest response predominantly from the saccule for downward movements. A significantly larger PPP value was recorded for the downward relative to upward movement for right (RTC onAA) and left (RTC onAA and RTC onBB) sides. For the left onAA and onBB movement a *p*-values <0.05 was obtained. The question remains unanswered as to whether an increased excitatory response (increased AP size) also elicits an increased (perhaps consequential) PPP response. With the exception of the left (BGi-onAA) value the right and left sitting up/down motion PPP sizes are significantly different (*p* < 0.05) from background PPPs measured immediately prior to movement. Further, the left PPP response was 95% confidence different between tilt and RTC motions i.e., (RTC BGi–RTC onAA)–(BGi-onAA) and (RTC BGi–RTC onBB)–(BGi-onBB) even accounting for any (BGi–RTC BGi) shift in PPP size. This is further evidence for a vestibular (otolithic) response.

Similarly, the supine up/down motion should evoke the strongest response from, predominantly, the utricle for the downward motion. A significantly larger PPP value for the downward relative to upward motion for right (RTC onAA) and left (RTC onBB) sides is observed. For the right onAA movement a *p*-values <0.05 is obtained. As above, the question remains unanswered as to whether an increased excitatory response (increased AP size) also elicits an increased (perhaps consequential) PPP response. With the exceptions of the right (BGi-onAA) and left (BGi-onBB) values, the right and left supine up/down motion PPP sizes are significantly different (*p* < 0.05) from background PPPs measured immediately prior to movement. Further, the right response is 95% confidence different between tilt and RTC motions i.e., (RTC BGi–RTC onAA)–(BGi-onAA) even accounting for any (BGi–RTC BGi) shift in PPP size. This again is further evidence for a vestibular (otolithic) response.

The horizontal rotation should evoke the strongest response for a rotation towards the recorded ear. A significantly larger PPP value is recorded on the right for both the right rotation and the RTC rotation, i.e., away from the right ear. There were no significant left side PPP changes. The arguments applied above to explain horizontal SCC AP responses can be equally applied to these discrepancies. There is a lack of clarity in the horizontal SCC data.

The vertical distance from PPP to AP was analysed (Figs. 11e and 11f) and showed the same patterns of significant differences seen in Figs. 11a–11d for up/down motions. Both the right and left onAA PPP to AP responses were 90–95% confidence different between tilt and RTC motions, i.e., (RTC BGi–RTC onAA)–(BGi-onAA) and (RTC BGi–RTC onBB)–(BGi-onBB) even accounting for any (BGi–RTC BGi) shift in PPP to AP size.

### Comparison of Nerve Deaf and Labyrinthectomy Responses with Normal

Patient 1 was nerve deaf in the right ear, while the left ear had normal hearing and he did not have any vestibular problems. Figure 12 shows the SP/AP plots of the patient 1s both ears. We assume the predominant recorded signals are acoustic and vestibular (muscle movement was minimised, no ECG is seen, eyes are closed, whilst recording was taken in a sound and electromagnetic signal attenuated chamber). For this patient, the right side is “pure” vestibular because the subject was nerve deaf on the right side (note wider AP and lack of pre-potential in supine position). The left side’s response shows a probable mix of vestibular and acoustic response (note the narrower AP) as expected.

**Figure 13:**
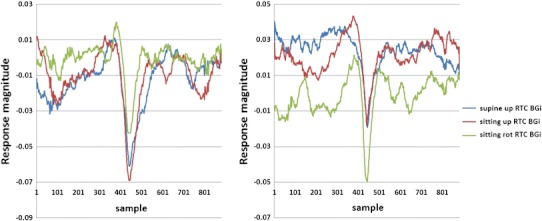
The RTC BGi SPAP plot recordings from patient 2 with normal left side (left plot) vestibular function and the right side chemical Labyrinthectomy (right plot). Left and right sides both had high frequency hearing losses

Patient 2 had a chemical Labyrinthectomy on his right side, while his left side vestibular was normal. Figure 13 shows the SP/AP plot of the RTC BGi recording for this patient. The plots (Figs. 13a and 13b) demonstrate a clear acoustic response component. The right side response is “pure” acoustic (note the narrower AP), and the left side shows a mix of vestibular and acoustic response (note the wider AP).

**Figure 14:**
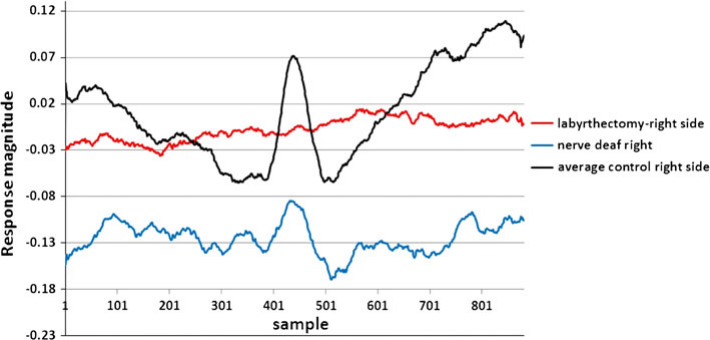
A comparison of right side sitting up RTC BGi–RTC onAA response curves (not normalised) for patient 1 with right side chemical Labyrinthectomy with left side normal (both left and right sides had a high frequency hearing loss) and for patient 2 with right side nerve deafness and left side normal. The black curve shows the average of 30 controls’ right side recordings

Figures 12 and 13 together clearly identify acoustic and vestibular components in the recording. The plots in Fig. 14 imply that following a Labyrinthectomy there is very little change in activity in response to a vestibular tilt. However, with nerve deafness the vestibular tilt evokes a response not dissimilar to the controls group.

### EMG Sources

**Figure 15:**
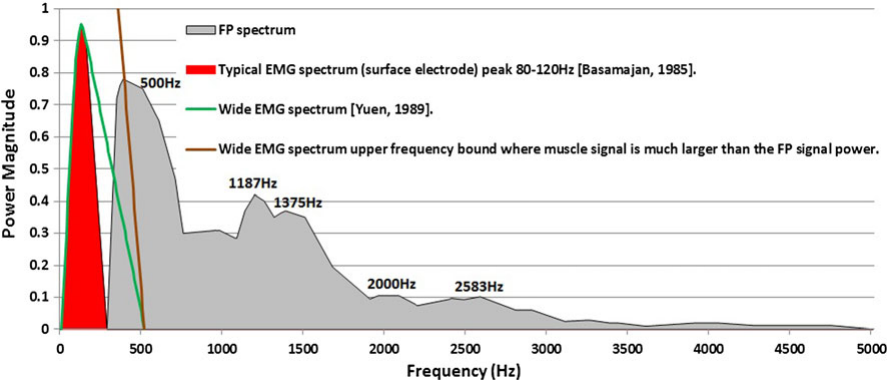
Power spectra for FP and EMG signals

The active electrodes are close, in particular, to the middle ear muscles the tensor tympani and stapedius. Figure 15 shows the spectrum for the simulated FP and overlayed is the spectrum of a typical (red)^2^ and wide bandwidth (green)^26^ EMG signals. The peak of the EMG signal is approximately between 80 and 120 Hz,^2^,^26^ which is much lower than the FP main peak (500 Hz). The brown curve is the case when the muscle’ signal power is significantly higher than the FP signal power, a likely scenario, and one where the EMG bandwidth is assumed to be wider.^26^ This represent a worst case scenario, while most of the FP spectra still remain outside the EMG spectrum. Given the NEER algorithm needs to detect sharp phase changes across the frequency ranges outside the EMG spectrum, it is expected that EMG corruption will exist but it would be unlikely to dominate the response. This was validated by high pass filtering the EVestG response with cutoff frequency of 350 Hz.

## Discussion

This study aimed to develop an algorithm for detecting spontaneous and driven FPs in the external ear canal. The particular objectives were: (1) to investigate whether the proposed NEER algorithm is able to detect FPs at negative SNRs with a high enough detection rate to enable generation of SP/AP like plots for vestibular signals; (2) to investigate whether the detected FPs are indeed a vestibular response to the particular movement stimuli and to investigate the potential separation of acoustic and vestibular signals by subtracting the resting response from the tilt response. The testing with simulated signals shows that the NEER algorithm is able to detect FPs in a noisy record. Despite the advantage of being able to detect the FPs in a very noisy record [down to (power) SNR = −30 dB], it is recognised that the major limitation of the algorithm is its low detection rate for FPs. In a negative SNR we will need many more FPs to average out the noise. However, the NEER method may be particularly useful in scenarios, where (1) the background noise can be well characterised, (2) there is a background signal that can be subtracted from the driven response wherein common uncorrelated signals can be eliminated, and (3) signal records are relatively long and there are numerous FPs. The case of recording the vestibular response to movement stimuli is indeed case 2, above.

The second objective was to investigate whether the averaged extracted SP/AP waveform output of the NEER algorithm is representative of the vestibular response particularly for the otolithic organs. This objective involved the potential separation of acoustic and vestibular signals by subtracting the resting response from the tilt response. Figure 14 shows a clear benefit from this subtraction. The results depicted in Fig. 11 are compatible with the known dynamics of vestibular physiology.^6^–^8^,^12^ We know that within the up/down (sitting and supine) motion stimulus the larger response change should be seen during the down versus up phase^20^ as shown in Figs. 11a–11f. If the size of the detected FP is correlated to the number of individual hair cells producing that FP, and/or to the degree of synchrony of hair cells’ firing to make that FP, then the AP size might be correlated with excitation^22^; therefore, it can be of diagnostic value.

There is very little response pattern difference observed between RTC BGi and RTC onAA for the combination that produced the largest difference up/down movement phases (Fig. 14) for the patient 2 with a Labyrinthectomy. Comparatively the nerve deaf patient produced a pattern comparable with that obtained from the control population. This is further evidence of a vestibular response component.

While it is beyond the scope of this paper to determine the exact mechanism(s) or exact origin(s) of these FPs, it has been possible to show clear acoustic and vestibular components in the EVestG outputs. EMG was discounted as a major source of corruption based on spectral separation and the NEER analysis method used (Fig. 15). Moreover, is has been possible to significantly differentiate the up and down responses for both supine up and up movements. For the AP point magnitude changes, this change may correspond to an increase in firing synchrony and a reduced random (spontaneous) firing component compared to that recorded in background (further discussion of this point is beyond the scope of this paper).

Oei *et* *al*.^20^ details a positive going response peak at a latency of 1.16 ms to an alternating vestibular driving stimulus. This occurs before the ECOG (and perhaps EVestG) response that has a known latency of about 1.9 ms.^9^,^10^ The pre-potential magnitude change may be related to this positive going response, or alternately may be due to some pre-potential hyperpolarisation. This is, however, difficult to interpret with the current experimental procedure that does not incorporate a repetitive driving stimulus.

The results of detected FPs in healthy controls show a clear and significant difference between static and dynamic responses in two of three independent movements as a result of analysis of the difference between the RTC BGi and RTC onAA SP/AP response plots. This is supportive of the hypothesis that by subtracting the two plots a curve more reflective of the vestibular response is obtained. This is emphasised in Fig. 14, wherein the vestibular movement change (RTC BGi–RTC onAA) following a Labyrinthectomy is severely diminished.

The SP/AP curve is still prone to noise corruption that may limit the number of detected FPs during each analysed 1.5 s segment. We can increase this duration by slowing the chair movement; however, that may reduce the dynamic vestibular response. Another plausible compromise would be to use repeated measures and combine the detected FPs from the 1.5 s segments to increase the number of detected FPs per segment but for this solution we have to be sure of the movement is exactly the same and initial conditions similar during the repeated measures. The ability to average multiple recordings is considered one path to improving the response accuracy. A compromise between recording time and efficacy is required. From the Introduction, the major limitation of acquiring up to 300 repeated vestibular recordings was time, typically more than 300 min, allowing at least 1 min for each whole body tilt. By being able to detect and analyse FPs recorded without the need for repeated stimulus evoked responses recording the time has reduced to 1 min for each tilt orientation thus making this method clinically applicable. The averaging of multiple recordings will be investigated in future studies as will the detailed impact of age on response.

## Conclusion

The NEER algorithm, despite having a low detection rate, appears the only algorithm currently available to effectively detect both spontaneous and tilt evoked individual acoustic/vestibular FPs in negative SNR recordings. When these averaged spontaneous and tilt responses are compared an index of vestibular function was generated. Vestibular responses to whole body movements were found to be directionally (up vs. down) significantly different and in agreement with known vestibular physiology. Further, there was some evidence to indicate these responses were different within the acceleration and deceleration phases of the up/down movement.

## Abbreviations

ABR: Auditory brainstem response
ECOG: Electrocochleography
EEG: Electroencephalogram
EMG: Electromyogram
ENG: Electronystagmography
EOG: Electrooculogram
FP: Field potential
NEER: Neural event extraction routine
RTC: Return to center
SCC: Semi circular canal
SNR: Signal-to-noise ratio
TDA: Time domain analysis
VEMP: Vestibular evoked myogenic potential
VsEPs: Vestibular evoked potentials
